# Mike Edmond on the value of change, learning, and not letting others set the agenda

**DOI:** 10.1017/ash.2023.436

**Published:** 2023-08-31

**Authors:** Michael Edmond

**Affiliations:** Chief Medical Officer, West Virginia University Health System, Morgantown, WV, USA

## Edited for clarity and brevity

**You’ve combined careers in medical education, infection prevention, leadership, and eventually became chief quality and safety officer, and later, chief medical officer. How did that unfold, and what inspired you to pursue these unique career paths?**

Well, I think I’m kind of unusual in that I decided that I wanted to do infectious diseases when I was a second-year medical student and then decided I wanted to be a hospital epidemiologist as a third-year medical student. I was goal directed and really pushed myself toward that path. There wasn’t formalized training in hospital epidemiology when I was a house officer, so I decided independently that the path to becoming a hospital epidemiologist was to do an ID fellowship and a master’s in public health. Therefore, I went to the University of Pittsburgh for ID fellowship and an MPH; however, late in my fellowship I realized that I still didn’t know the first thing about hospital epidemiology. I asked my attendings at UPMC “here’s what I want to do; how do I accomplish that?” And virtually everybody had the same answer, which was, “there’s this guy in Iowa and he’s *The Guy,* so if this is really what you want to do you should probably try to work with him.” They were of course referring to Dick Wenzel.

I typed a letter and sent it to Dr. Wenzel, and he wrote me back inviting me to visit the University of Iowa. When I visited, I knew right away that this was the right next step for me, so I went there to pursue a fellowship in hospital epidemiology after I finished ID fellowship. On completing the hospital epidemiology fellowship, Dick asked me to stay on as faculty at Iowa and I did, but then at the end of my first year, he said, “I’m going to Virginia Commonwealth University to be the Chair of Medicine and I’d like you to go with me.”

I asked, “what do you want me to do there?” And he said, “I have 2 things I need you to do: be the director of the internal medicine residency program, and the hospital epidemiologist.”

I said, “Sure, when do I start?” I didn’t even visit Richmond and I’d never been there in my life. I just showed up a day before I was supposed to start because he’s a great guy and I trusted him implicitly.

From day one of my first real job, I’ve been at least 50% administrative. I learned from my time as chief resident that I enjoy administrative work and organizing things, which some find either burdensome or boring. Hospital epidemiology and overseeing the residency program were a good fit for this reason.

When I arrived at VCU, the residency program needed a lot of help to get “on its feet,” which took me about 5–6 years. I was successful, but also realized that once the program stabilized, I wasn’t as interested, because the organizing and building part was over. What I enjoyed the most about it was taking something that was broken and putting it back together.

As I was doing heavy administrative work, I realized that I didn’t have any formal training in administration, and so I decided to get a master’s in public administration, which was a great experience; however, if I were to do it over, I would have obtained an MBA instead. My problem was that I never wanted to know about money. In college, I never took economics because I saw myself as a future doctor and felt that discussing money was “dirty.” However, I realized over time that it was a significant hole in my knowledge.

Several things happened next. There was a new chair of medicine when Dick retired, which was difficult for me. Things were changing and I was becoming more unhappy. I decided that I needed to find a new place and role which would offer a growth experience. I was fortunate for the opportunity to go back to the University of Iowa as the Chief Quality Officer.

It was a big step in terms of broadening the scope of my responsibilities, and I really like change and learning, which is why I’ve been in school most of my life. I eventually obtained an MBA a few years after getting settled in Iowa and it was fascinating. It was one of the best experiences I’ve had in my career. I recommend it to anybody who aspires to an administrative role and seeks leadership skills. Every single course was fascinating, and my only regret is not pursuing it sooner.

**Did that set you up for being the Chief Medical Officer, in your opinion?**

Yes, it seemed to be the next most logical step for me. A week before I finished my MBA, I got an email about the CMO position in West Virginia. I grew up about 30 minutes from Morgantown and had gone to medical school there. It was all very familiar to me, but I never really thought about coming back. When I came to visit, I realized how much was going on here, and how much growth and positive energy there was, and it really excited me. So, I am now the Chief Medical Officer for the University Hospital, the health system, and our two physician practice plans. The system has 25 hospitals in 4 states, with 6 billion dollars in revenue and 4,000 providers.

**Why do you think so many physicians are resistant to embracing change and what advice do you have for them?**

I think that physicians in general are creatures of habit and tend to be ritualistic. They get into their way of doing things. Change is hard for everybody, but I think harder for physicians because of their nature. As a leader, you must build trust with people so you can allay their fears of change.

**You’ve served as a mentor to many after being mentored by Dick Wenzel. What did Dick provide as a mentor and what is the secret to your success in mentoring so many individuals?**

In the modern era, Dick was one of the first people that really had an interest in hospital epidemiology and infection prevention. When I first moved to Iowa, I didn’t know what to expect. There were 16 epidemiology fellows—it was huge, and they were from all over the world. Didier Pittet was there along with Trish Perl, and many others. It was like the “who’s who” of hospital epidemiology. There was so much energy with many people interested in the same issues. Part of Dick’s success was his ability to build this program. He’s just a great guy and a wonderful human being. Dick is a very positive person, and he never foresees barriers, so anything is possible in his mind, and that is very attractive to people. This is the kind of person you want to hang out with, who has these big ideas and doesn’t see any reason why they can’t be achieved. He is also a very nurturing person, and his personality is very motivational, so you want to please him and do even more. It’s this sort of positively reinforcing cycle of good things happening when you work with Dr. Wenzel.

**You’ve been very successful as a mentor as well, Mike. What’s the key to your success?**

I don’t think I’ve really been a mentor, rather, I’ve been more of a guide for people on their career path. I’ve had the great fortune to work with lots of young people who are very bright.

**Let’s switch gears and talk about your “Controversies in Hospital Infection Prevention” blog.** ^1^ **Tell us about that experience and what made it exciting.**

In 2003, there was a lot of controversy about vaccinating healthcare workers against smallpox. The Iraq war and bioterrorism threats were happening, and there was a directive that healthcare workers should be vaccinated against smallpox. I didn’t think that was a very good idea from an infection prevention standpoint because of all the immunosuppressed patients in the hospital and the potential to transmit the vaccine virus to them. It was something that worried Dick and me.^2^ Nobody really knew what to do, but there was no forum for hospital epidemiologists to discuss this and so everybody made the decision to vaccinate or not in isolation. I felt there was a real need for hospital epidemiologists to have some way of communicating about issues that are current. Sometime after that, Trish Perl started an email group that is still active today. Finally, you could get some sense of where the community was on these issues.

Over a few years, I did a lot of work with our media relations people, and one of them said to me, “you know, you really ought to start a blog addressing some of these controversial issues.” I was like, “oh, that’s an interesting thought,” but didn’t really have any knowledge of how to do that. In 2009, SHEA met in San Diego. Dan Diekema and I were having dinner there and had this discussion about starting a blog covering the many controversial issues in hospital IP, which lacked a real discussion forum. We thought it would be a cool thing to do, but I still logistically didn’t know where to begin. Then, a week after SHEA, I got an email from Dan stating that he started the blog, and it just took off from there. We published a lot of posts and for years had something new to say almost every day. It was probably one of the most rewarding things I’ve done because I really enjoy writing and creatively exploring controversial topics in real time. It really helped me in a lot of ways in the 10 years we kept it going. It also resulted in multiple lecture invitations and several publications. It also forced me to stay on top of critical issues in the field and better prepare for my own job as hospital epidemiologist at VCU at the time. It was fun to have people come up to me after a presentation and share how much they enjoyed reading the blog. One time I remember complaining to a CDC person about an issue I felt they were not addressing despite its importance to the hospital epidemiology community. He said, “Why don’t you blog about it? You know, everybody at CDC reads your blog. So, if you write about it, they’ll see it.”

**Did you ever have any misadventures with the blog?**

Looking back, I don’t think I ever wrote anything that was problematic. I think there was only one blog piece that I ever took down. My feeling was that if I wrote it, I’m going to stand behind it and I’ll leave it out there.

**You have over 200 publications. Which are the most meaningful to you?**

Objectively, the most important article I wrote was on the descriptive epidemiology of nosocomial bloodstream infections,^3^ which has been cited 5,600 times and counting, although it’s over 20 years old. I don’t think there was anything earth-shattering in that article. However, there are a few articles that are important to me personally, like one about racial bias in the selection process of internal medicine residency candidates.^4^ It got no attention at the time; in its first 10 years it was cited 15 times, but in the last 5 years it’s been cited over 100 times, so I am gratified that it has added recent value to the conversation about bias in academic medicine.

The other one is a piece that I wrote called “Taylorized Medicine,” which was an opinion piece about the transformation occurring in residency programs due to ACGME changes primarily around duty hour rules.^5^ I reflected on the loss of the joy of being an internist due to the transformation. Personally, it was important to be able to write that.

**As a leader, how do you approach difficult decisions and tough conversations. Do you have a philosophical approach to them?**

I make lots of decisions and some of them are difficult. I think the most difficult decisions revolve around people. Other decisions are primarily data based (e.g., volume, cost, acquisitions, etc.) and are generally easier to make. Conflict resolution requires active listening to consider all sides of the issue. Decision making in these instances is driven by your values and your vision for the future. We all encounter setbacks or challenges that may threaten to move our careers in a direction that we don’t desire. What I try to tell people is that as painful as things are in the moment, these challenges can turn into something greater. Leaving VCU was incredibly painful; I never anticipated leaving a place I thought I would work until I retired. It didn’t work out the way I desired, but in retrospect, it moved me into a whole different sphere and forced me to grow into new opportunities. Unanticipated challenges turn into great growth opportunities, but you don’t always see that in the present moment. It takes time to look back and say, “hey, you know, that turned out great.”

**What in your opinion are important topics or challenges to focus on over the next 5–10 years?**

In Infectious Diseases, there are always new pathogens and increasing drug resistance in the old ones. But what we need more than anything in hospital epidemiology is a deeper understanding of how to prevent transmission. Years ago, the biggest controversy in the field revolved around contact precautions. Surprisingly, we’re still having the same debate. Prevention of transmission is the core of hospital epidemiology, right? And yet, I feel as if we’ve still not figured it out. It’s not all our fault, though, because it takes funding to do that, and hospital epidemiology research has never been adequately funded. Moreover, (and this is a generalization), the typical hospital epidemiologist is introverted, very hard working, effective, but most comfortable working away from the spotlight, and doesn’t take credit for their good work. The dominant personality type in our field allows for other people to have a louder voice. So, I guess my primary criticism is that hospital epidemiologists have not been effective in setting the agenda for infection prevention. They’ve allowed other people to do it. The blog was one way to try to influence that, and I think it probably did.

**Tell us about the books and articles on your nightstand.**

I almost exclusively read non-fiction, though I am very slowly reading *The Covenant of Water* by Abraham Verghese (I think every ID doctor must read his books). I mostly read books that are helpful to me in my administrative work or leadership roles. One is a book called *Humbitious: The Power of Low-Ego, High-Drive Leadership*, which describes how the best leaders are both humble and ambitious. In the last month, I’ve read 2 books that are somewhat related. The first one is called *STFU: The Power of Keeping Your Mouth Shut in an Endlessly Noisy World*. It teaches how to talk less and be more effective. The other book is Smart Brevity, which teaches how to communicate effectively in a world of people with short attention spans.

**Any final thoughts for our readers?**

At this moment, I am, in both my personal and professional lives, exactly where I want to be. It has taken a great deal of work and time to get here, but I’m incredibly grateful to have reached this point.
